# Screening for Depression Using Natural Language Processing: Literature Review

**DOI:** 10.2196/55067

**Published:** 2024-11-04

**Authors:** Bazen Gashaw Teferra, Alice Rueda, Hilary Pang, Richard Valenzano, Reza Samavi, Sridhar Krishnan, Venkat Bhat

**Affiliations:** 1 Unity Health Toronto St. Michael’s Hospital Interventional Psychiatry Program Toronto, ON Canada; 2 Toronto Metropolitan University Department of Computer Science Toronto, ON Canada; 3 Toronto Metropolitan University Department of Electrical, Computer, and Biomedical Engineering Toronto, ON Canada; 4 University of Toronto Department of Psychiatry Toronto, ON Canada

**Keywords:** depression, natural language processing, NLP, sentiment analysis, machine learning, deep learning, transformer-based models, large language models, cross-cultural, research domain criteria, RDoC

## Abstract

**Background:**

Depression is a prevalent global mental health disorder with substantial individual and societal impact. Natural language processing (NLP), a branch of artificial intelligence, offers the potential for improving depression screening by extracting meaningful information from textual data, but there are challenges and ethical considerations.

**Objective:**

This literature review aims to explore existing NLP methods for detecting depression, discuss successes and limitations, address ethical concerns, and highlight potential biases.

**Methods:**

A literature search was conducted using Semantic Scholar, PubMed, and Google Scholar to identify studies on depression screening using NLP. Keywords included “depression screening,” “depression detection,” and “natural language processing.” Studies were included if they discussed the application of NLP techniques for depression screening or detection. Studies were screened and selected for relevance, with data extracted and synthesized to identify common themes and gaps in the literature.

**Results:**

NLP techniques, including sentiment analysis, linguistic markers, and deep learning models, offer practical tools for depression screening. Supervised and unsupervised machine learning models and large language models like transformers have demonstrated high accuracy in a variety of application domains. However, ethical concerns related to privacy, bias, interpretability, and lack of regulations to protect individuals arise. Furthermore, cultural and multilingual perspectives highlight the need for culturally sensitive models.

**Conclusions:**

NLP presents opportunities to enhance depression detection, but considerable challenges persist. Ethical concerns must be addressed, governance guidance is needed to mitigate risks, and cross-cultural perspectives must be integrated. Future directions include improving interpretability, personalization, and increased collaboration with domain experts, such as data scientists and machine learning engineers. NLP’s potential to enhance mental health care remains promising, depending on overcoming obstacles and continuing innovation.

## Introduction

### Background

Depression is a prevalent mental health disorder affecting 280 million people worldwide and accounts for more than 47 million disability-adjusted life-years [1,2]. Characterized by symptoms such as persistent feelings of sadness, diminished interest, and impaired daily functioning, depression can severely impact an individual’s quality of life [3] and is associated with suicide and premature mortality from comorbidities [4]. However, traditional methods of diagnosing and screening for depression primarily rely on subjective clinical assessments, which can be time-consuming and susceptible to the inherent biases of health care professionals [5].

In recent years, researchers have explored advanced technologies like natural language processing (NLP) to address the limitations of traditional methods in detecting and understanding depression [6,7]. NLP, a field of artificial intelligence (AI), enables machines to automatically analyze and extract valuable insights from textual data [8]. This technology shows immense potential in transforming how we identify and manage mental health disorders. By leveraging the vast amount of digital information generated daily, including social media posts, electronic health records, and web-based forums, NLP can assist in detecting subtle linguistic cues and patterns that may indicate depressive symptoms [9].

The integration of NLP in detecting depression offers multiple advantages. Not only does it promise improved accuracy and efficiency, but it also brings the advantage of scalability. By analyzing large amounts of textual data on a population level, NLP enables a comprehensive understanding of depression, potentially leading to early detection and intervention. In addition, NLP-based approaches might contribute to the reduction of the stigma surrounding mental health [10] by providing a more objective and nonjudgmental assessment of individuals’ emotional well-being. This shows that NLP can help create a more supportive environment for those dealing with depression.

However, while NLP shows significant potential for mental health care, we must also recognize the challenges and ethical considerations that come with its implementation. Issues like privacy concerns, data security, and potential biases demand critical analysis. In this literature review, we aim to explore the current state of research on using NLP techniques for detecting depression. We will discuss the successes and limitations of this rapidly evolving technology, along with its future scenarios in improving mental health diagnosis and care. By shedding light on this rapidly evolving field, our goal is to foster informed discussions and encourage further advancements that will ultimately benefit individuals living with depression while promoting more effective mental health screening systems.

### This Review

This literature review aimed to provide a broad overview of the potential and challenges of using NLP for depression screening by extracting valuable information from textual data. While we discuss various NLP techniques and their applications, the focus is on presenting a comprehensive view of the field rather than delving into technical details. Our goal is to offer readers a high-level understanding of the opportunities and limitations presented by NLP in detecting depression while also highlighting ethical considerations and future directions. By providing this broad perspective, we hope to foster further exploration and innovation in applying NLP to enhance mental health support systems.

## Methods

### Literature Search Strategy

A comprehensive literature search was conducted using 3 web-based databases—Semantic Scholar, PubMed, and Google Scholar. These databases were chosen for their extensive coverage of research in the fields of computer science, health care, and AI. The search aimed to identify studies focusing on depression screening using NLP techniques.

The search strategy involved using a combination of relevant keywords and Boolean operators. The following search terms were used: “depression screening” OR “depression detection” AND “natural language processing” OR “NLP.” This search query was tailored to each database to ensure compatibility with their specific search functions.

The inclusion criteria for selecting studies were broad, encompassing a range of study designs, including original research articles, review papers, and technical reports. Studies were included if they discussed the application of NLP techniques for depression screening or detection, addressed the successes and limitations of such approaches, or explored ethical considerations and potential biases. No restrictions were placed on the publication date to ensure a comprehensive overview of the historical development and current state of the field.

### Study Selection and Data Extraction

The initial search yielded many results. To manage the screening process efficiently, the titles and abstracts of the retrieved studies were imported into reference management software (Zotero). Duplicates were removed, and the remaining studies were screened.

During the initial screening, studies were assessed based on their relevance to the research topic. Studies that did not specifically address depression screening or detection using NLP were excluded. Studies focusing solely on other mental health disorders without a clear connection to depression were also excluded.

In the study selection process, 2 independent reviewers initially screened titles and abstracts against the predefined inclusion and exclusion criteria. Discrepancies between the reviewers were identified and resolved through a consensus meeting, where both reviewers discussed their decisions and clarified any misunderstandings related to the criteria. If consensus could not be reached, a third independent reviewer was consulted to provide an additional perspective and make the final decision. The full texts of the remaining studies were then reviewed in detail by the 2 independent reviewers, following the same process described for the title and abstract screening. Studies were included in the final selection if they provided substantial contributions to the understanding and application of NLP in depression screening. This included discussions on NLP techniques, depression detection methods, classification models, datasets, ethical considerations, cross-cultural perspectives, or future directions in the field.

Data extraction was performed concurrently with the full-text review. Relevant information from each study was extracted and organized into a structured format. This included details, such as the study’s main objectives, methodologies used, key findings, limitations, and potential future directions suggested by the authors. The extracted data were then synthesized and analyzed to identify common themes and gaps in the literature, forming the basis for the discussion section of this literature review.

This review aimed to provide an up-to-date overview of the field by following the literature search strategy. It highlights the potential and challenges of using NLP for depression screening, along with ethical considerations and future research directions.

### Task Definition and Scope

#### Overview

The primary task addressed in this literature review is the detection or screening of depression using NLP techniques. This task involves the automatic analysis of textual data, such as social media posts, electronic health records, or clinical interview transcripts, to identify indicators of depressive symptoms and provide a classification or assessment of an individual’s mental health status.

The scope of this review encompasses various subtasks and aspects related to depression detection, including the following: (1) classification, (2) severity classification, (3) depressive symptoms identification, (4) risk assessment, and (5) personalized depression analysis.

#### Classification

This involves categorizing textual data into depressive or nondepressive states, often using supervised machine-learning algorithms. The goal is to accurately distinguish between individuals experiencing depression and those who are not.

#### Severity Classification

Beyond binary classification, some studies focus on assessing the severity of depressive symptoms. This involves categorizing depression into different levels or stages, such as mild, moderate, or severe, based on the linguistic cues present in the text.

#### Depressive Symptoms Identification

NLP techniques are used to identify specific depressive symptoms, such as negative emotion, persistent feelings of sadness, changes in cognitive processes, or expressions of hopelessness. This task helps in understanding the nuanced emotional and cognitive states associated with depression.

#### Risk Assessment

Some studies aim to go beyond detection and focus on assessing the risk of depression-related outcomes, such as suicide risk or the likelihood of developing major depressive disorder. This task involves analyzing linguistic cues that may indicate a higher risk for adverse events.

#### Personalized Depression Analysis

There is a growing interest in personalized depression analysis, where NLP techniques are used to tailor interventions and treatments to individuals. This involves identifying unique linguistic patterns and behaviors associated with specific subgroups of depressed individuals.

By outlining these tasks and scope, we provide a clear framework for the literature review and ensure that the discussion remains focused on the application of NLP in depression detection and related areas.

## Results

### Overview

**Figure 1:**
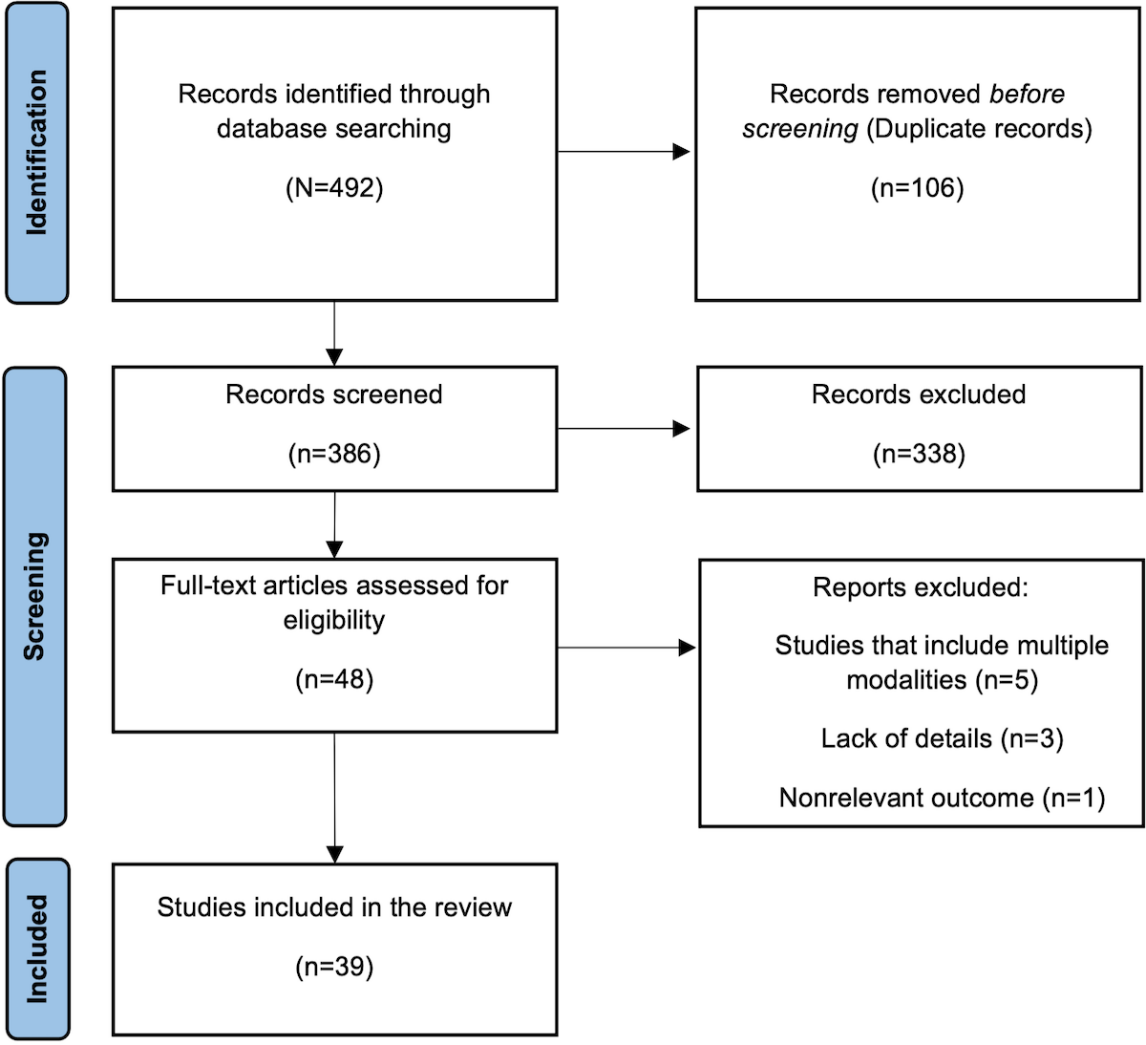
PRISMA (Preferred Reporting Items for Systematic Reviews and Meta-Analyses) flow diagram.

The literature search strategy described in the Methods section returned a diverse range of studies focusing on various aspects of depression detection using NLP techniques. These studies spanned different methodologies, including original research, review articles, and technical reports. The key findings from these studies focusing on the NLP techniques are summarized in Table 1 below and presented in a structured format for clarity. The PRISMA (Preferred Reporting Items for Systematic Reviews and Meta-Analyses) flow diagram is presented in Figure 1 and the PRISMA checklist is presented in Multimedia Appendix 1.

**Table 1:** Summary of natural language processing (NLP) techniques for depression detection.

NLP techniques	Methodology	Relevance to depression detection	Limitations	Study	Sample size, n	Dataset	Method	Results
Sentiment analysis	Analyzes the emotional tone of the text	Identifies negative language	Limited understanding of depression	Rathner et al [11], 2017	220 participants	Recruited participants were asked to reflect on their past year	LIWC^a^ based features	*R*^2^ value of 0.104
Sentiment analysis	Analyzes the emotional tone of the text	Identifies negative language	Limited understanding of depression	Prabhu et al [12], 2022	189 sessions	DAIC-WOZ^b^	Word2vec feeds them as input to long short-term memory	82.3% accuracy
Linguistic markers	Identifies linguistic features related to depression	Captures cognitive distortions and identifies the use of certain types of words	May overlook contextual complexities	Islam et al [13], 2018	7145 comments	Facebook user comments	Decision tree classifier from feature obtained through LIWC	*F*-measure of 0.71
Linguistic markers	Identifies linguistic features related to depression	Captures cognitive distortions and identifies the use of certain types of words	May overlook contextual complexities	De Choudhury et al [14], 2021	554 users	Twitter	LIWC for determining 22 specific linguistic styles	72.4% accuracy
Word embedding	Creates vectorized word representations	Preserves semantic relationships	May miss nuanced semantics	Stankevich et al [15], 2018	887 users	CLEF^c^ and eRisk 2017	Word embeddings and support vector machine model	*F*_1_-score of 63.4%
Word embedding	Creates vectorized word representations	Preserves semantic relationships	May miss nuanced semantics	Lopez-Otero et al [16], 2017	189 sessions	DAIC-WOZ	GLoVe^d^ vector inputs	*F*_1_-score of 73%
Word embedding	Creates vectorized word representations	Preserves semantic relationships	May miss nuanced semantics	Mallol-Ragolta et al [17], 2019	189 sessions	DAIC-WOZ	GloVe embeddings	Unweighted average recall of 0.66
Word embedding	Creates vectorized word representations	Preserves semantic relationships	May miss nuanced semantics	Dinkel et al [18], 2020	189 sessions	DAIC-WOZ	Pretrained word embeddings (ELMo^e^)	*F*_1_-score of 84%
Word embedding	Creates vectorized word representations	Preserves semantic relationships	May miss nuanced semantics	Rutowski et al [19], 2020	16,000 sessions	American English spontaneous speech	GloVe word embedding	AUC^f^ of 0.8
Convolutional neural networks and recurrent neural networks	Captures local and sequential information in language data	Models language patterns of depressed individuals	Complex architectures, data-hungry	Korti and Kanakaraddi [7], 2022	—^g^	Twitter	Recurrent neural network with long short-term memory	91% accuracy
Convolutional neural networks and recurrent neural networks	Captures local and sequential information in language data	Models language patterns of depressed individuals	Complex architectures, data-hungry	Tejaswini et al [20], 2022	13,000 posts	Reddit and Twitter	Fasttext with long short-term memory	87% accuracy
Large language models	Captures complex linguistic nuances and context	Achieves high-level understanding	Computationally expensive and requires specific fine-tuning	Senn et al [21], 2022	189 sessions	DAIC-WOZ	Fine-tuning BERT^h^ and its variants	*F*_1_-score of 0.62
Large language models	Captures complex linguistic nuances and context	Achieves high-level understanding	Computationally expensive and requires specific fine-tuning	Hayati et al [22], 2022	53 participants	Interview questions	Few-shot learning on GPT^i^-3	*F*_1_-score of 0.64
Large language models	Captures complex linguistic nuances and context	Achieves high-level understanding	Computationally expensive and requires specific fine-tuning	Németh et al [23], 2022	Approximately 80,000 posts	Data acquired through SentiOne	Fine-tuning DistilBERT	73% precision

^a^LIWC: Linguistic Inquiry and Word Count.

^b^DAIC-WOZ: Distress Analysis Interview Corpus–Wizard-of-Oz set.

^c^CLEF: Conference and Labs of the Evaluation Forum.

^d^GLoVe: global vectors for word representation.

^e^ELMo: embeddings from language models.

^f^AUC: area under the curve.

^g^Not available.

^h^BERT: bidirectional encoder representations from transformers.

^i^GPT: generative pretrained transformer.

### Historical Timeline of NLP Development and Relevance of Depression

NLP has gone through substantial development over the years, with technological advances and research contributing to its growth. Throughout its history, the field of NLP has continually expanded its capabilities, and the relevance of depression screening within this timeline has become increasingly noticeable.

#### Early Years and Rule-Based Systems (1950s-1970s)

The origins of NLP can be traced back to the 1950s with the development of early computer programs like the Georgetown-IBM Experiment (developed jointly by Georgetown University and IBM) [24], which attempted to translate Russian sentences into English using basic rules and structures. In the 1970s, rule-based systems gained prominence. Systems like SHRDLU [25] demonstrated limited language understanding by manipulating blocks in a virtual world based on user commands. However, these systems had difficulty handling the complexity of natural language, including expressions of emotions and sentiment.

#### Knowledge-Based Approaches and Syntax Analysis (1980s-1990s)

In the 1980s, there was a shift toward knowledge-based approaches, including expert systems [26]. Researchers attempted to encode linguistic rules and world knowledge to improve language understanding. In the 1990s, statistical methods, such as the hidden Markov model (HMM) [27] and part-of-speech tagging [28], gained popularity. These methods improved parsing and syntactic analysis, but the understanding of context, semantics, and emotions in language remained challenging.

#### Machine Learning and Statistical Methods (2000s-2020s)

Machine learning algorithms, such as support vector machine and multi-layer perceptron improved performance on several NLP tasks, including sentiment analysis. Deep learning models, such as recurrent neural networks (RNNs) and convolutional neural networks (CNNs), later enhanced NLP. Word embeddings like Word2Vec and global vectors for word representation (GloVe) captured semantic relationships between words. Attention mechanisms and transformers, used in models like bidirectional encoder representations from transformers (BERT) and generative pretrained transformer (GPT), achieved remarkable results in language understanding and generation. These methods in relation to depression will be explained in detail in the Classification Models for Depression Detection: Machine Learning and Current State of the Art Models in Depression Detection sections.

#### Relevance of Depression in the NLP Timeline

With the growth of web-based platforms and social media, textual data became abundant, and there was increased interest in the application of NLP for sentiment analysis and mood detection. Early efforts to identify emotional states and linguistic markers of depression emerged. The deep learning revolution then enabled more nuanced sentiment analysis and emotion recognition. Researchers started to explore the detection of mental health conditions, including depression, using NLP techniques. Studies focused on extracting linguistic cues related to depressive symptoms and emotional states from text data. Specific methods of depression detection using NLP will be further discussed in the following sections.

### NLP Techniques for Depression Detection

NLP techniques have been shown to be important in obtaining valuable insights from textual data acquired through social media, web-based forums, or textual health records for depression detection [13,20]. Given certain textual data, NLP can convert—through multiple steps—the textual data into a format that can point toward the presence or absence of depression. Among the initial steps in depression detection through NLP, *text preprocessing* and *feature extraction* play an essential role. Text preprocessing involves converting text data into a structured format suitable for analysis. Researchers have used techniques like tokenization, stemming, and lemmatization to achieve this [12]. Tokenization breaks down a certain transcript into individual words or tokens (which are parts of words). Stemming and lemmatization are both processes that involve reducing words to their base or root forms. Stemming often uses chopping (eg, jumps → jump, caring → car), while lemmatization applies language and context analysis for accurate reductions (eg, better → good, caring → care).

Another NLP technique that has been used to convert text into a more usable representation that has led to an increase in the accuracy of depression detection is feature extraction from text. Some examples of these methods include bag of words and term frequency-inverse document frequency [6]. The bag of words method creates a count-based representation of words present in a certain text by treating each word as a separate unit and, therefore, ignoring the order of the words. Term frequency-inverse document frequency assigns weights to words based on their count in a document and across the entire dataset, giving more importance to rare but distinctive words. These methods enable researchers to transform raw textual data into a quantitative representation, which can be used for further processing down the road.

One of the most widely used NLP techniques, and one that has been used as a proxy for identifying depression in language is *sentiment analysis* [29]. This approach examines the emotional tone of a text. Prior studies have shown a higher correlation between individuals with depression and the use of more negative words and the frequent expression of emotions related to sadness and hopelessness in their written language [30]. The linguistic inquiry and word count tool [31] is one example of such a sentiment analysis technique. This tool enables the automatic analysis of texts into preset word categories associated with depression—including negative emotions and cognitive processing [11]. By quantifying the emotional expressions in text data, researchers can gain valuable insights into the emotional state of individuals, which can in turn point toward the identification of potential depressive symptoms.

Researchers have also made substantial efforts to identify *linguistic markers and cues* that capture patterns related to depression. For example, prior studies have revealed that certain linguistic features, such as an increased use of first-person pronouns and a decreased use of third-person pronouns, can indicate the presence of depression [14,32]. In addition, the presence of cognitive distortions, characterized by negative thinking, has been found in the language of individuals with depression [33]. This suggests that by examining language patterns, NLP techniques offer a window into the cognitive and emotional processes underlying depression.

**Figure 2:**
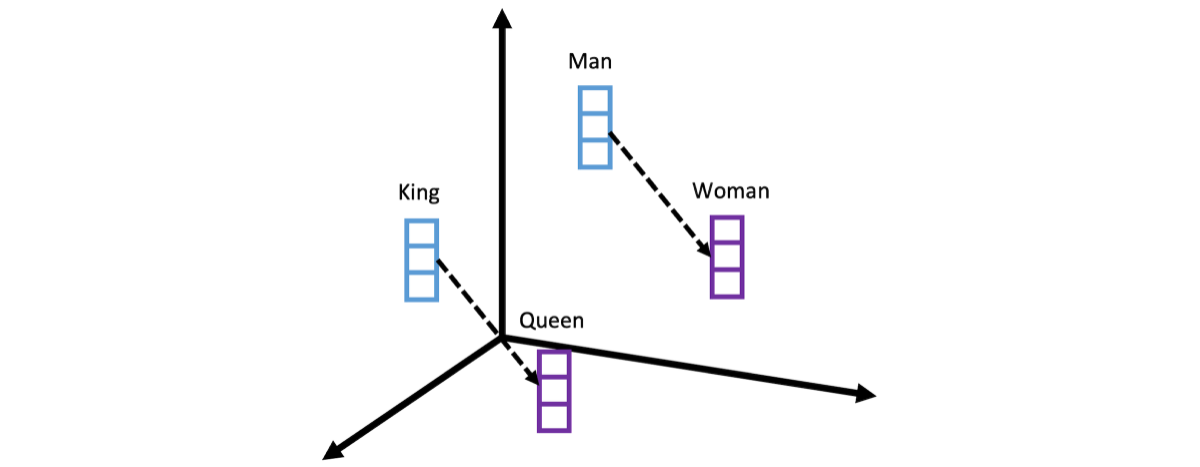
Visualization of how word embeddings capture analogy information from the words.

Recent advancements in NLP have enhanced the field of mental health disorder detection in general, especially using vectorized representations of language. *Word embeddings* and *contextual analysis using deep learning models* have substantially improved the accuracy and performance of depression detection models [34-37]. Word embeddings are created by transforming words into continuous vector representations, capturing semantic relationships and contextual meaning between words. To grasp the concept of word embeddings, envision a vector space where words are positioned based on their semantic meanings, allowing for intriguing relationships like “king” – “man” + “woman” = “queen” (see Figure 2 for the visualization of this analogy). Static word embeddings, such as GloVe [37], transform words into fixed vector representations that capture global semantic relationships, while dynamic embeddings, like embeddings from language model (ELMo) [38], provide context-dependent word representations. In the context of depression-related text data, previous studies have leveraged both GloVe [15-17] and ELMo [18] embeddings to capture word semantics and have achieved better accuracy in depression detection tasks. By preserving semantic relationships, word embeddings enable NLP models to better understand the meaning of words in context, which enhances the capacity to identify linguistic indicators of depression.

In summary, the use of NLP techniques, such as sentiment analysis, linguistic markers, and recent advancements like word embeddings contribute to a powerful toolkit for detecting depression from the text. These techniques have enabled researchers and clinicians to gain valuable insights into an individual’s mental health state through their written and spoken languages, potentially giving rise to more accurate detection and intervention strategies for depression and other mental health disorders. As research in this domain continues to evolve, combining the strengths of classic NLP with cutting-edge developments—which will be described in the coming section—promises to enhance the understanding of depression further, leading to improved mental health outcomes for individuals worldwide.

### Classification Models for Depression Detection: Machine Learning

Machine learning models have emerged as a powerful tool in depression and mental health disorder detection in general, offering good capabilities to classify depressive and nondepressive states accurately. Leveraging the large amount of digital text data generated daily, these models have the potential to enhance mental health care by enabling more efficient and objective approaches to identifying and managing depression.

One approach is supervised machine learning where various algorithms have been applied to depression detection tasks, particularly binary classification (depressed or nondepressed) based on linguistic features extracted from text. Logistic regression, support vector machines, random forests, naive Bayes, and multi-layer perceptron classifiers are among the commonly used models, and they have shown promising results in accurately identifying and classifying depressive states [11,23,39]. These models use labeled datasets to learn patterns and relationships between textual features and depression status, contributing to more accurate and robust predictions.

Another set of approaches are the unsupervised machine learning techniques which have been used to uncover hidden structures within the depressed population. Techniques, such as K-means and hierarchical clustering, aim to identify distinct subgroups based on their linguistic patterns [40]. By grouping individuals with similar language use together, these clustering approaches have the potential to uncover different characteristics of depression. Such insights could lead to the development of personalized treatment strategies, catering to the unique needs of subgroups within the larger depressed population.

The introduction of deep learning has further advanced depression detection in the scope of NLP. For example, in the context of depression detection, CNNs and RNNs have gained popularity for their ability to capture sequential information and model temporal dependencies in language data [7,20]. CNNs effectively analyze local patterns within a text, while RNNs are well-suited for understanding the contextual dependencies that arise from sequential data. By capturing the linguistic cues related to depression, these deep learning architectures have substantially increased the performance of depression detection models from prior machine learning models. For example, Tejaswini et al [20] developed a novel approach called Fasttext convolution neural network with long short-term memory to detect depression in social media text data obtained from Reddit and Twitter. Their method achieved an 87% accuracy in distinguishing depression from nondepression in a dataset comprising of 13,000 samples, highlighting its potential for early detection of depressive states.

### Current State of the Art Models in Depression Detection

Transformer-based large language models (LLMs), another class of deep learning models, have also shown exceptional results in mental health detection tasks [41]. These models, characterized by their substantial number of parameters, often in the hundreds of millions to trillions, are artificial neural networks designed for natural language understanding and generation, using a self-attention mechanism to process input data in parallel and capture contextual information across long sequences. The transformer architecture [42] has been widely used in language modeling tasks and has proved to be highly effective in sentiment analysis [29,43-45]. In the context of depression detection, these models excel at understanding the complex and nuanced emotional language used by individuals experiencing depression [21,22,39].

**Figure 3:**
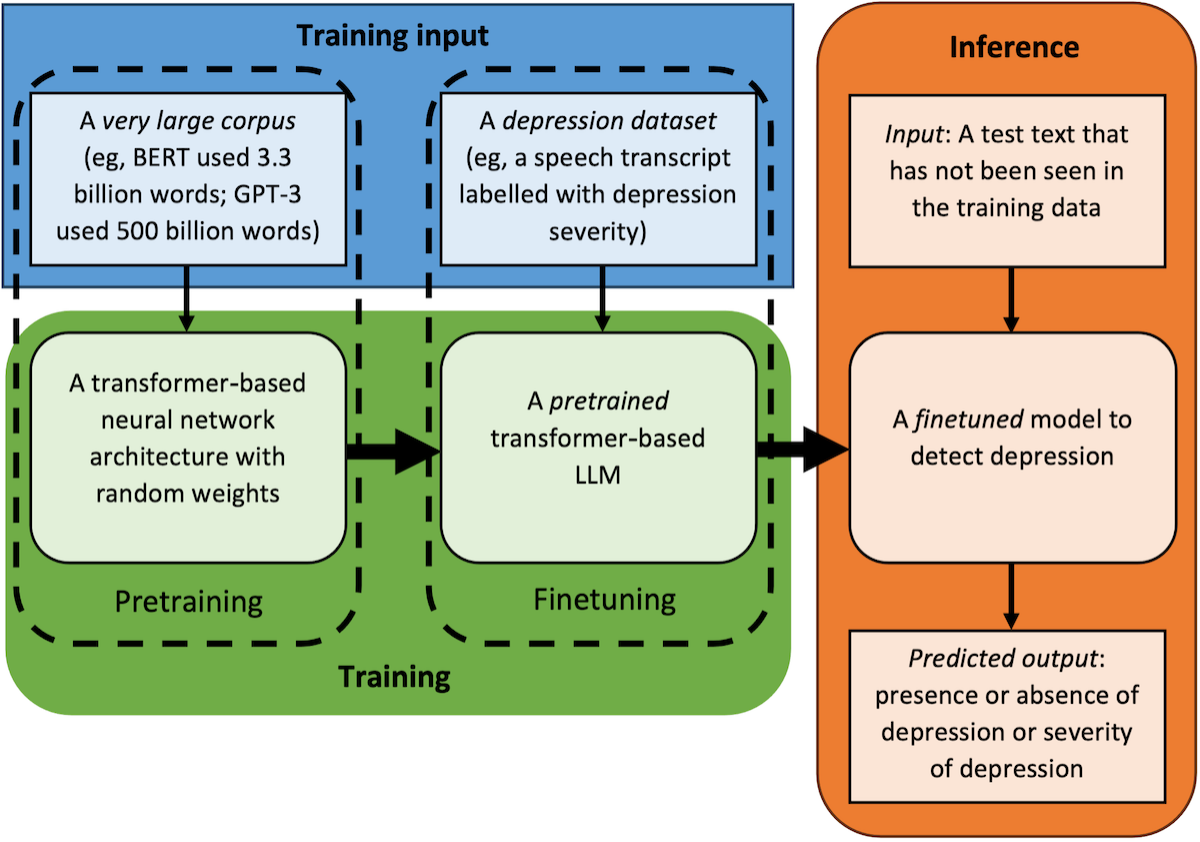
Steps to generate a typical large language model (LLM)–based depression detection model. BERT: bidirectional encoder representations from transformers; GPT: generative pretrained transformer.

One of the essential attributes of LLMs in the context of depression detection lies in their ability to do transfer learning—a technique that involves pretraining a model on a large and comprehensive dataset before fine-tuning it for a specific task. This paradigm has exhibited immense potential in the domain of depression detection [19]. Notable pretrained language models, such as BERT [46], MentalBERT [47], and GPT [48] have been fine-tuned on datasets curated from depression-related text sources. This strategic adaptation enables these models to grasp the details of the lexicon and linguistic context unique to depression [21,22]. The outcome is a refined model capable of discerning and contextualizing the language manifestations that accompany depression, thereby enhancing both accuracy and generalizability in depression detection models. Figure 3 shows the typical steps taken to generate a fine-tuned model from a pretrained model to make an inference and predict depression.

By pretraining on vast and diverse linguistic datasets, these LLMs develop an innate comprehension of the structures and nuances of human language. This foundational knowledge enables them to decode the unique linguistic markers and emotional cues exhibited by individuals struggling with depression. The understanding of context enables these models to not only identify obvious expressions of depressive thoughts and emotions but also to identify the subtle and sometimes ambiguous linguistic signals that often evade traditional diagnostic approaches.

In summary, incorporating machine learning and deep learning models into depression detection has not only enhanced accuracy but also opened new avenues for understanding and managing mental health disorders. The ability of these models to identify patterns, relationships, and distinct subgroups within the depressed population offers valuable insights for early detection, personalized treatment, and more effective mental health interventions. As research in machine learning and deep learning continues to advance, the potential for further improving depression detection and mental health care becomes increasingly promising. Using the power of these technologies responsibly will be critical in realizing their full potential in enhancing mental health care and supporting individuals affected by depression. Table 1 summarizes the different NLP techniques discussed above.

### Datasets Used for Depression Detection

The availability of appropriate datasets plays a vital role in training and building NLP models, including for tasks, such as depression detection. In recent times, several publicly accessible datasets, which include a depression label, have emerged [49-55]. Some of the datasets used for depression detection are presented in Table 1.

One of the most popular datasets is the “distress analysis interview corpus/wizard-of-Oz set” [51]. This dataset consists of audio (which includes transcript) and video recordings that simulate clinical interviews designed to assess distress levels related to depression, anxiety, and posttraumatic stress disorder. During these interviews, some participants act out distress, showcasing symptoms and experiences associated with depression, while others take on the role of interviewers, referred to as the “Wizard-of-Oz.” Researchers use this dataset to develop and evaluate computational methods and algorithms for automatic distress detection and analysis, specifically focusing on cues related to depression-related distress. In another dataset, as presented in the study by Matcham et al [56], they presented data collected from 623 participants with a history of recurrent major depressive disorder. The study used smartphone sensors, wearable devices, and app-based questionnaires over 11 to 24 months. In addition, speech data were collected every 2 weeks through a speech task involving predetermined text and open-ended responses, with 82.2% of participants providing speech data.

However, despite these advancements, certain characteristics of the datasets present challenges when it comes to the trained models’ ability to generalize. For example, small and imbalanced datasets (as observed in Jamil et al [55], where only 5% of the tweets contained a reference to depression) can cause underfitting or overfitting, such that the model becomes too tailored to the training data, making it less effective in handling new and unseen data [57]. In addition, the lack of diversity in the data, with most of the samples originating from specific demographics, may limit the applicability of the models across different populations. These limitations highlight the need for continuous improvement in dataset curation and selection to enhance the performance and applicability of depression detection models.

### Validation and Evaluation Metrics

Evaluating NLP models for depression detection involves carefully selecting appropriate metrics to assess their performance. In this context, some commonly used quantitative evaluation metrics include accuracy, precision, recall, *F*_1_-score [58], and the area under the receiver operating characteristic curve [59]. The results of different studies using these metrics can be seen in Table 1. Researchers often use cross-validation and external validation strategies to ensure the models’ generalizability and effectiveness.

However, being mindful of the potential limitations and biases associated with these quantitative metrics and validation techniques is essential. For instance, while accuracy is a commonly used metric, more informative evaluations metrices are needed for imbalanced datasets—where one class significantly outweighs the others. The model may achieve high accuracy in such cases by classifying most samples into the majority class [60], but might not reflect the actual performance of the prediction model.

Cross-validation techniques [61], which are widely used for model evaluation, also have limitations that need to be considered. One common approach involves splitting the dataset into 2 parts, using one for training the model and the other for testing its performance. However, this method may introduce higher bias, as crucial information from the unutilized data is left out during the training phase.

Another cross-validation technique, leave-one-out cross-validation, attempts to mitigate bias by training on the entire dataset while sequentially leaving out one data point for testing. Although this approach uses all data points, it can lead to higher variation in the testing phase, mainly if the omitted data point is an outlier. In addition, the leave-one-out cross-validation method demands substantial execution time as it iterates over the number of data points, making it computationally expensive for large datasets.

Alternatively, researchers can opt for k-fold cross-validation, where the dataset is divided into k subsets, and the model is trained on all except one subset reserved for evaluation. This approach helps reduce bias compared with simple two-part cross-validation, but it can still introduce variation in the testing phase due to the different subsets used for evaluation.

As researchers use cross-validation to assess NLP models for depression detection, they must be mindful of these limitations and biases. Ensuring accurate and reliable model assessment requires understanding the trade-offs of each method and carefully selecting the most appropriate validation technique based on the dataset’s characteristics and research objectives. By being aware of these considerations, we can ensure more robust and trustworthy evaluations of NLP models in the crucial domain of depression detection.

Aside from quantitative metrics, qualitative evaluation metrics also play a substantial role in assessing the performance of NLP models for depression detection, as they offer insights beyond numerical measurements. While quantitative metrics provide valuable information about the models’ accuracy and efficiency, qualitative evaluation metrics explore the models’ interpretability, user experience, and overall impact.

One important qualitative evaluation metric is interpretability [62]. NLP models, especially those using complex deep learning techniques, are often considered “black boxes” because it is challenging to understand how they arrive at their predictions. However, interpretability is essential in applications related to mental health. Clinicians, researchers, and users must comprehend how the model reaches its conclusions to trust its decisions. Therefore, techniques that explain the model’s predictions, such as attention mechanisms or feature visualization [63], are essential for ensuring the model’s transparency and interpretability.

User experience and acceptability are also crucial qualitative metrics to consider. When deploying NLP-based depression detection systems in real-world settings, it is vital to measure end users’ experiences—such as patients and therapists. Feedback from users can shed light on the system’s usability, ease of integration into existing workflows, and its ability to provide valuable insights during the prediction. Exploring user perspectives can help improve the model’s design, its practicality, and effectiveness in real-world mental health settings.

Ultimately, qualitative evaluation metrics complement quantitative assessments by offering a more comprehensive understanding of the NLP model’s impact on mental health care. By considering interpretability and user experience, we can develop more well-rounded and effective NLP-based depression detection systems that align with the needs and expectations of both patients and mental health professionals.

### Cultural and Multilingual Perspectives

The cultural and linguistic diversity surrounding mental health expressions presents distinctive challenges when it comes to detecting depression across different languages and cultures [64,65]. Researchers have recognized these challenges and sought to address them through cross-cultural research, highlighting the necessity for culturally sensitive depression detection models [66].

An illustrative example of such efforts is the study conducted by Lyu et al [67]. In this research, the focus was on depression detection using text-only social media data from the Chinese platform Weibo. The researchers considered a broader range of linguistic features that are relevant to depression, considering cultural factors and suicide risk specific to the Chinese language. To achieve this, they analyzed depression scores and past posts from 789 Weibo users. The outcome was a predictive model that showed promising results in detecting depression among the Chinese-speaking population.

This study served as an important reminder of the need for cultural expressions when it comes to improving the recognition of depression within specific linguistic and cultural groups. By considering the unique ways in which individuals from different languages and cultural backgrounds communicate their mental health experiences, we can improve the accuracy of depression detection models and make them more inclusive. As we continue to explore and expand our understanding of cross-cultural and multilingual perspectives on depression detection, we can move toward providing better mental health support and care for diverse populations around the world.

## Discussion

### Principal Findings

This literature review explored the potential of NLP in enhancing depression screening by analyzing textual data. The review revealed a range of NLP techniques, including sentiment analysis; linguistic markers; word embeddings; and deep learning models, such as CNNs, RNNs, and LLMs, that have been successfully applied to depression detection. The studies demonstrated the efficacy of these techniques, with machine learning models achieving high accuracy in classifying depressive states. However, ethical concerns, including privacy, bias, and interpretability, were also identified as critical challenges. In addition, the importance of cross-cultural and multilingual perspectives was emphasized, highlighting the need for culturally sensitive models. The review further discussed the integration of depression detection using NLP within the research domain criteria (RDoC) framework, mapping linguistic cues to psychological and biological constructs. Overall, the findings showcase the potential of NLP in enhancing mental health support systems while also presenting ethical and technical challenges that require continued innovation and collaboration.

### Comparison to Prior Work

The main findings of this review align with prior work in the field, which has also identified the potential of NLP techniques in mental health detection [9,10,29]. However, this review extends the understanding by incorporating the latest advancements in LLMs and their application to depression detection. Previous studies have largely focused on traditional machine learning techniques, while this review highlights the significant potential impact of deep learning and LLMs, as well as the comparison of current state-of-the-art models with prior classification models for depression detection.

### Integration of Depression Detection Using NLP Within the RDoC Framework

The RDoC framework provides a comprehensive approach to investigating mental health and psychopathology by focusing on fundamental psychological and biological systems rather than relying solely on traditional diagnostic categories. The RDoC framework acknowledges the complexity of mental health conditions and aims to foster new research approaches that lead to improved diagnosis, prevention, intervention, and treatment [68]. Here, we will explore how the detection of depression using NLP aligns with the principles and objectives of the RDoC framework.

The RDoC framework is organized into several major functional domains, each containing psychological and biological dimensions or constructs that span the range from normal to abnormal functioning [68]. Depression, a multifaceted mental health condition, touches upon multiple domains within the RDoC framework. These domains include negative valence systems, positive valence systems, cognitive systems, and social processes. NLP techniques for depression detection often analyze textual data to extract linguistic cues, sentiment, and cognitive patterns related to depression [14,29,30,32]. These patterns can be mapped onto constructs within the RDoC domains to gain insights into the underlying psychological and biological mechanisms associated with depressive symptoms.

For example, persistent negative emotions, such as sadness, hopelessness, and irritability, often characterize depression. This can be mapped to the negative valence systems domain in the RDoC framework. Some NLP methods that practice sentiment analysis for depression detection use linguistic markers associated with negative emotions and cognitive distortions [29,30]. Similarly, the cognitive systems domain in the RDoC framework focuses on cognitive processes and depression often involves cognitive distortions. NLP techniques can identify linguistic markers indicative of cognitive distortions within a text, connecting cognitive processes and linguistic expressions in depression [33].

Depression can also be mapped to the social processes domain within the RDoC framework which encompasses constructs related to social behavior, social cognition, and social communication. NLP methods offer insights into individuals’ social expressions and interactions through their textual data, including social media posts and web-based forum discussions [20].

### Ethical Considerations and Limitations in NLP for Depression Detection

As NLP holds great potential in enhancing depression detection, it also gives rise to a range of ethical concerns that require careful consideration. Mental health data are highly sensitive. The information shared by individuals could include personal experiences, emotions, and medical history. Therefore, one of the main concerns includes safeguarding privacy and security of mental health data. To ensure the protection of individuals seeking support through NLP-based mental health services, it becomes crucial to use robust privacy and security measures. For privacy, even if data are anonymized, there may be a risk of reidentification. Clinical notes provide significant contextual information (eg, favorite movies and sports) that could be used by adversaries to be linked back to some other background information (eg, age group, general location information, etc) and identify the individual. Privacy regiments for NLP-based models, in addition to anonymization, should include guaranteed statistical privacy through differential privacy [69]. For security, in addition to cryptographic protection of data storage with support for provable worst-case security, an authentication and authorization regimen should be put in place to ensure the prevention of accidental or intentional unauthorized access.

Further ethical challenges arise from the potential biases that NLP models may inherit from the training data, giving rise to concerns regarding the robustness of depression detection [70-72]. Lack of robustness can have several adverse impacts when NLP is deployed in analyzing mental health text. A poor NLP model generalization (the distribution shift from the data the model is trained with to the data used in deployment) may lead to the model’s failure to generalize well across various linguistic styles, terminologies, or expressions used by individuals. Lack of contextual understanding is another challenge as it might lead to the model losing essential contextual information, making it challenging to understand the full spectrum of an individual’s mental state. Finally, bias and fairness issues are important aspects of model robustness. For instance, if certain demographic groups are underrepresented in the training data, it can lead to reduced accuracy for those specific populations, causing inequalities in health care.

In addition to these concerns, another substantial challenge arises from the integration of third-party application program interfaces and cloud-based services in NLP-based depression detection. While using third-party application program interfaces can enhance the capabilities of NLP models by, for example, incorporating pretrained language embeddings, it introduces a layer of dependency and potential security risks. These risks come from the need to share sensitive mental health data with external services, raising questions about data ownership, use, transparency, and compliance with privacy regulations.

A recent study by Straw et al [70] shed light on the integration of AI in health care. It underscored the critical need for collaboration between computer scientists and medical professionals to address biases in NLP models used in psychiatry. The research involved a comprehensive literature review of NLP applications in mental health, specifically evaluating biases in GloVe [37] and Word2Vec [36] word embeddings. The findings revealed significant biases related to religion, race, gender, nationality, sexuality, and age. Moreover, the review highlighted the need for more attention to these biases in existing research, signaling a limited cross-disciplinary collaboration in this domain.

Straw et al [70] emphasized the importance of addressing biases to prevent health gaps caused by AI and data-driven algorithms. They offered valuable recommendations for future research to minimize potential harm. By proactively working to identify and mitigate biases in NLP models, we can strive to create more equitable and just mental health support systems, ensuring that the benefits of NLP technology are accessible and effective for all individuals, regardless of their background or demographic characteristics. Textbox 1 summarizes some of the challenges and opportunities in using NLP for detecting depression.

**Challenges and opportunities in natural language processing for depression detection.**

**Challenges and opportunities**

- Privacy concerns: robust anonymization techniques
- Biases: transparency and fairness
- Interpretability: explainable artificial intelligence
- User feedback: user-centric design
- Cross-cultural variations: culturally sensitive models
- Small, imbalanced datasets: data diversity

### Limitations of the Study

While this review provides valuable insights into the application of NLP for depression screening, some limitations should be acknowledged. First, the scope of the literature search was confined to 3 databases: Semantic Scholar, PubMed, and Google Scholar. Although these sources cover a wide range of academic publications, the exclusion of other major databases like IEEE Xplore and Scopus may have resulted in the omission of relevant studies, particularly those focusing on technical advancements in NLP. This may limit the breadth of the findings and leave certain innovations in NLP techniques underrepresented.

In addition, this review relies on qualitative synthesis without performing a meta-analysis of the selected studies. A quantitative meta-analysis could have provided a more robust statistical evaluation of the effectiveness of different NLP techniques in depression screening. Furthermore, while ethical and cross-cultural issues were discussed, the review does not deeply analyze how these concerns are addressed across different studies. This omission could overlook critical gaps in ensuring the fairness and applicability of NLP models across diverse populations, limiting the generalizability of the findings.

### Future Directions

Despite the substantial potential that NLP has shown in depression detection, several challenges still lie ahead. The ambiguous characteristics of natural language and the ever-changing nature of language use in diverse contexts make it difficult to achieve consistently accurate detection. In addition, the risk of overfitting on small datasets and the necessity for large-scale, diverse datasets to ensure robust model training remain pressing concerns [41].

Future studies should focus on tackling these challenges to advance the field of NLP in depression detection. One important avenue for exploration is improving the interpretability of NLP models. As complex deep learning techniques become prevalent, it becomes increasingly crucial to understand and explain how these models arrive at their predictions. For example, the use of LLMs for interpretable mental health analysis has been explored in studies like that of Yang et al [73]. These models aim to provide not just predictions but also explainable insights into the mental health status of individuals. This direction holds the potential for improving the trustworthiness and clinical applicability of LLMs in depression screening. Future research should continue to address the challenges of accuracy, ethical considerations, and collaboration between NLP experts and mental health professionals to fully realize the benefits of LLMs in this context.

Personalization is another crucial aspect that future research should address. Depression is a complex and highly individualized condition, and so a one-size-fits-all approach may not be sufficient to meet the variable needs of individuals. Developing personalized depression detection tools that consider an individual’s unique linguistic pattern, behavior, and context can enhance the accuracy of the detection process. These tools can cater to specific user requirements and provide tailored support and interventions.

Integrating user feedback and domain expertise in developing NLP models to achieve these advancements is crucial. Engaging with end users, including patients and mental health professionals, can provide valuable insights into the strengths and limitations of the models and help refine them to suit real-world applications better. Collaborating with domain experts, such as psychologists and psychiatrists, can ensure that the models align with clinical practices and address the most relevant aspects of depression detection and treatment.

Overall, the future of NLP in depression detection holds significant potential, but it also demands continued innovation and collaboration. By addressing challenges, improving interpretability, and personalizing depression detection tools, we can pave the way for more effective, user-centric, and clinically relevant solutions that contribute substantially to mental health care.

### Conclusions

In conclusion, adopting NLP techniques for depression detection holds significant potential in enhancing mental health support systems. This literature review has explored various NLP methodologies, applications, and challenges in detecting depression using textual data. While obstacles remain, ongoing advancements in NLP, ethical considerations, and cross-cultural insights pave the way for more accurate, accessible, and equitable mental health solutions.

The evolving field of NLP offers the potential for more effective detection of depression, but challenges persist. Overcoming the complexity of natural language and obtaining diverse datasets are key focus areas. Ethical considerations underscore the need for data privacy and model transparency. Integrating cross-cultural insights ensures culturally sensitive solutions that cater to diverse populations. With continued progress and collaboration, NLP can improve mental health care and well-being worldwide.

## Appendix

Multimedia Appendix 1 PRISMA (Preferred Reporting Items for Systematic Reviews and Meta-Analyses) checklist.

## Abbreviations

AI: artificial intelligence
BERT: bidirectional encoder representations from transformers
CNN: convolutional neural network
ELMo: embeddings from language model
GloVe: global vectors for word representation
GPT: generative pretrained transformer
HMM: hidden Markov model
LLM: large language model
NLP: natural language processing
RDoC: research domain criteria
RNN: recurrent neural network
